# Fertility and family planning in Uttar Pradesh, India: major progress and persistent gaps

**DOI:** 10.1186/s12978-019-0790-x

**Published:** 2019-08-23

**Authors:** Shiva S. Halli, Damaraju Ashwini, Bidyadhar Dehury, Shajy Isac, Antony Joseph, Preeti Anand, Vikas Gothalwal, Ravi Prakash, B. M. Ramesh, James Blanchard, Ties Boerma

**Affiliations:** 1 Department of Community Health Sciences Faculty of Medicine, University of Manitoba, 750 Bannatyne Avenue, Winnipeg, Manitoba R3E 0W3 Canada; 2 India Health Action Trust, VK Commerce, Rajajinagar Industrial Area, Rajajinagar, Bangalore, Karnataka 560044 India

**Keywords:** Total fertility rate, Family planning, Geographical variation, Uttar Pradesh, NFHS

## Abstract

**Background:**

Uttar Pradesh (UP) is the most populous state in India with historically high levels of fertility rates than the national average. Though fertility levels in UP declined considerably in recent decades, the current level is well above the government’s target of 2.1.

**Data and methods:**

Fertility and family planning data obtained from the different rounds of Sample Registration System (SRS) and the National Family Health Survey (NFHS). We analyzed fertility and family planning trends in India and UP, including differences in methods mix, using SRS (1971–2016) and NFHS (1992–2016). Bivariate and multivariate regression analyses were used.

**Results:**

From 2000, while the total fertility rate (TFR) declined in UP, it is still well above the national level in 2015–16 (2.7 vs 2.18, respectively). The demand for family planning satisfied increased from 52 to 72% during 1998–99 to 2015–16 in UP, compared to an increase from 75 to 81% in India. Traditional methods play a much greater role in UP than in India (22 and 9% of the demand satisfied respectively), while use of sterilization was relatively low in UP when compared to the national averages (18.0 and 36.3% of current married women 15–49 years in UP and India, respectively in 2015–16).

Within UP, district fertility ranged from 1.6 to 4.4, with higher fertility concentrated in districts with low female schooling, predominantly located in north-central UP. Fertility declines were largest in districts with high fertility in the late nineties (B = 7.33, *p* < .001). Among currently married women, use of traditional methods increased and accounted for almost one-third of users in 2015–16. Use of sterilization declined but remained the primary method (ranging from 33 to 41% of users in high and low fertility districts respectively) while condom use increased from 17 and 16% in 1998–99 to 23 and 25% in 2015–16 in low and high fertility districts respectively.

**Conclusions and implications:**

Greater reliance on traditional methods and condoms coupled with relatively low demand for modern contraception suggest inadequate access to modern contraceptives, especially in district with high fertility rates. Family planning activities need to be appropriately scaled according to need and geography to ensure the achievement of state-level improvements in family planning programs and fertility outcomes.

## Plain English summary

There has been a substantial drop in fertility in Uttar Pradesh. The comparative trends between India and Uttar Pradesh show that the Total Fertility Rate (TFR) in the state has always remained above the national average but the fertility trajectory is similar. In fact, the fertility decline in Uttar Pradesh (UP) has accelerated since 2000, and the fertility gap between India and UP has reduced substantially. However, there are substantial variations in the TFR across districts of Uttar Pradesh, ranging from 1.6 to 4.4. When the districts were classified into different clusters based on their fertility levels, this formed a geo-spatial clustering of regions with different levels of fertility. For instance, Tarai belt and its adjacent districts in Central UP formed the cluster with high fertility region and Bundelkhand region was the low fertility cluster.

The fertility decline in UP was driven by an increase in use of contraceptive methods among married women. Female sterilization was the predominant method in UP but the percent of married women sterilized was only half as prevalent as in India. Condom use was the second most popular method and has an increasing trend, and is twice as prevalent as in India. The use of traditional methods (Rhythm/Periodic Abstinence and Withdrawal) of family planning, most notably periodic abstinence, was more than two times higher in UP compared to India. To achieve government TFR target, activities need to be appropriately scaled and targeted according to need and geography to ensure the achievement of state-level improvements in family planning programs and fertility outcomes.

## Introduction

India has experienced a dramatic fertility decline over the last 50 years, from 5.2 children per woman in 1971–72 to 2.2 children by 2015–16 [1, 2]. The fertility decline occurred in almost all sections of society- rich and poor, literate and non-literate, upper caste and lower caste, Hindus and Muslims [3–5]. Improvements in child mortality, women’s schooling, and economic development are considered important contributing factors to reductions in desired family size and increased use of modern contraceptive methods (Contraceptive Pills, Intrauterine contraceptive devices, Injectable contraceptive, Female Sterilization, Male Sterilization, Lactational Amenorrhea, female/male condom, Foam/Jelly & Standard Days Methods) [6–9]. The use of modern contraceptives among married women 15–49 years increased from 31.7 to 47.8% between 1992 and 93 and 2015–16, even though there are concerns about lack of increase since the 2005–06 [10]. Subnational differences in fertility and family planning by state and district also persist despite the secular decline in Total Fertility Rate (TFR) [11].

In 2000, Government of India formulated the National Population Policy to achieve a TFR of 2.1 by 2012 and population stabilization by 2045 by addressing family planning unmet needs [12]. The extent to which these national targets are met primarily depends on progress in India’s states with large populations. The fertility transition began in south India, reaching replacement level fertility of 2.1 as early as 1990, especially in Kerala and Tamil Nadu states [13, 14] and same trend was subsequently followed by states like Maharashtra, Punjab, Himachal Pradesh, Jammu and Kashmir, eastern states of West Bengal and Odisha [15]. However, fertility rates in several states in the north-central India remained well above replacement level, where the slow pace of fertility decline especially in states with large populations has partly been attributed to lagging socio-economic development [16].

Fertility levels in Uttar Pradesh (UP), the most populous state in India having 199.8 million populations [17], are well above India’s national rate. Despite the fact that UP experienced a fertility decline during the past few decades from 1992 to 2015, the TFR in the state is still above the replacement level and 20% higher than the national estimate (2.7 and 2.18 respectively) according to the most recent round of NFHS (2015–16).

This paper examines the fertility decline and district differences in UP to understand current progress and identify areas that need to be addressed to sustain or accelerate the fertility decline. We first compared the fertility decline in UP to India as a whole with a focus on changes in the coverage of contraceptive methods and demand met for family planning. We then analyzed fertility and family planning in the administrative districts of UP to identify populations with high fertility and low contraceptive use, which has implications for future efforts to meet the need of all couples in UP through policies and programs.

## Methods

Data from different rounds of Sample Registration System (SRS) and National Family Health Survey (NFHS) were used. The SRS was established in 1964 to provide national and state level estimates of fertility and mortality [1]. The SRS includes continuous enumeration of vital events from a sample of villages/urban blocks. The data quality has been considered to be good [18]. We used TFR from SRS to assess fertility levels and trends.

The NFHS is a large-scale multi-round survey which is conducted in a representative sample of households throughout India. Four rounds of the survey have been conducted between 1992 and 93 and 2015–16. The surveys provide national and state information on fertility, infant and child mortality and practice of family planning along with other socio-economic characteristics. UP has undergone several administrative changes over the period of time, including geographical re-organization of district boundaries, formation of new districts and separation of a group of districts to form a separate state. Considering these changes, the NFHS trend analysis is limited to just three rounds of surveys (Rounds 2, 3 & 4). From the second NFHS (1998–99) survey we excluded districts of Uttarakhand state (formed from select districts of UP in 2000) from the UP district estimates.

For the district level fertility analysis within UP, we computed total fertility rates using the birth history data from women 15–49 years in NFHS-2 (1999–2000) and NFHS-4 (2015–16), using Bruno Schoumaker’s method (2013) [19]. The NFHS-1 (1992–93) and NFHS-3 (2005–06) data sets did not have comparable district level information. In UP, the survey took several years to complete and total fertility rates refer to different years depending upon the date of survey in that district.

The comparison of the fertility trend in UP with India focuses on the average annual rate of decline in fertility and FP methods by time-period. Within UP, we examined clustering of fertility using NFHS data in 2015–16 and assessed the extent to which district characteristics were associated with fertility: level of female education (36% of women did not attend school), proportion of population from poor wealth quintile including poorest, proportion who were Muslim (19%) and proportion of respondents belong to Scheduled Caste (23%). In addition, we assessed the extent to which the relative change in total fertility during 1998–99 and 2015–16 can be explained by district changes in schooling levels and urbanization, baseline levels of total fertility in 1998–99, religious and scheduled caste composition of the districts in 2015–16. Comparable data from the two NFHS surveys were available for 71 of the 75 districts; and for the newly formed four districts namely CSM Nagar, Shamli, Hapur and Sambhal the information was not available in NFHS-4. Bivariate and multivariate linear regression models were used to assess statistical significance.

For the analysis of contraceptive use patterns and trends, districts were grouped into three categories, namely, low (<= 2.3), medium (2.4–2.9) and high (> = 3.0) depending on NFHS-4 TFR levels. Similarly, based on rate of decline in TFR, two groups were formed out of total 71 comparable districts in NFHS 2 and 4, 36 districts belong to the faster decline cluster and 35 districts belong to the slower decline cluster. The rate of decline in TFR was calculated by estimating Total Fertility Rate and the percentage change using NFHS rounds 2 and 4 ((NFHS 2-NFHS 4)/NFHS 2 * 100). The criteria for deciding faster and slower rate of fertility decline is to have almost equal number of districts in each group given the small sample size. For each district, change in proportion was calculated between NFHS-3 and NFHS-4 for characteristics associated with fertility such as female education, urbanization, Muslim and Scheduled Caste populations. We examined the differences in coverage with modern and traditional (Rhythm/Periodic Abstinence and Withdrawal) family planning methods in NFHS 1998–99 and NFHS 2015–16. We used the family planning demand satisfied, which is a better coverage indicator as it measures the use of contraceptive methods among married women 15–49 years who need a contraceptive because they do not want to become pregnant and are exposed to the risk of getting pregnant. The demand for family planning satisfied with modern contraceptive methods is measured as the number of married women who use modern contraceptives divided by the sum of the number of married women whose need for family planning is not met (unmet need) and the number of married women who use modern contraceptives. The extent to which the demand for family planning is met for all methods (modern and traditional) is measured in the same way.

## Results

### Levels and trends in TFR: state and national level scenario

**Fig. 1:**
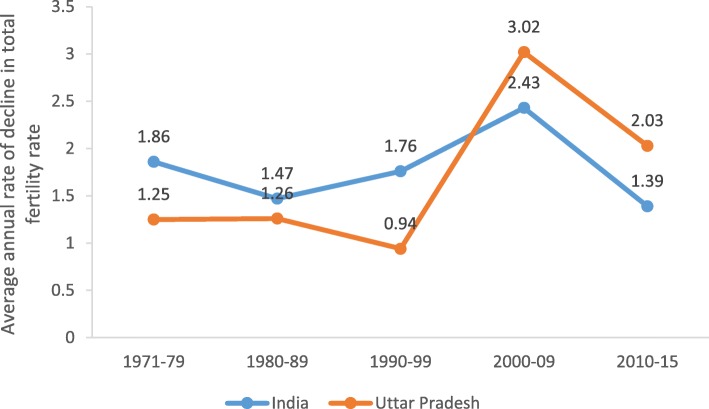
Average annual rate of decline in TFR in India and Uttar Pradesh, 1971–2016, India. Source: Sample Registration System (SRS)

The fertility decline in UP was slower than India during all decades until 2000 but exceeded the national pace of decline during 2000–2009 and 2010–2016 (0.6% per year faster decline), resulting in a narrowing of the gap between India and UP (Fig. 1). The TFR in UP of 2.7 in the NFHS 2015–16, however, remained much higher and above the Government of India’s target of 2.1 by 2012 and national average of 2.18 in 2015–16. Unmet need in UP was 18.0% compared to 12.9% in India in 2015–16 resulting in an estimated 8.2 million women with an unmet need for family planning.

**Fig. 2:**
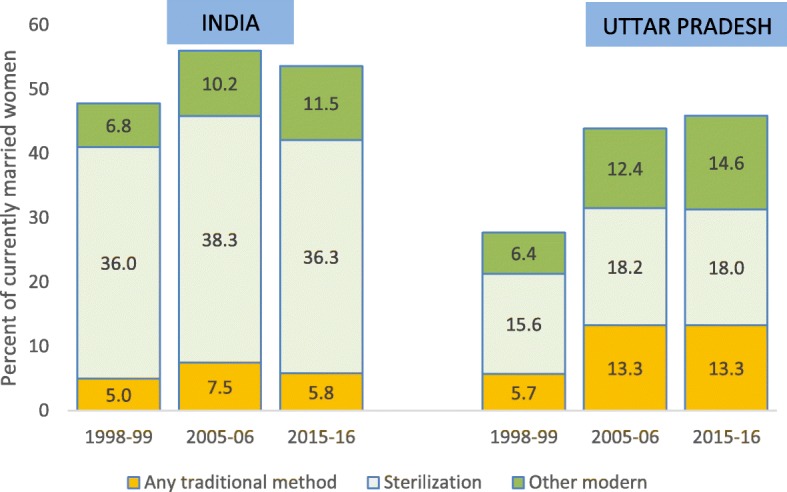
Contraceptive use among married women 15–49 years in India and Uttar Pradesh, 1998–2016. Source: National Family Health Surveys (NFHS)

Contraceptive use among married women 15–49 years differed markedly between India and UP (Fig. 2). First, the large gap in modern contraceptive use between India and UP declined since 2005–06 but remained substantial in 2015–16 (48.5 and 30.6% respectively). Second, while sterilization is by far the most popular method in India, the levels differ greatly with 36.3 and 18.0% of couples in India and UP respectively having one of the partners sterilized. Third, the use of traditional methods increased from 5.7 to 13.3% by 2015–16 in UP, almost entirely driven by greater reported use of periodic abstinence, and was more than twice as high as in India (5.8%).

**Fig. 3:**
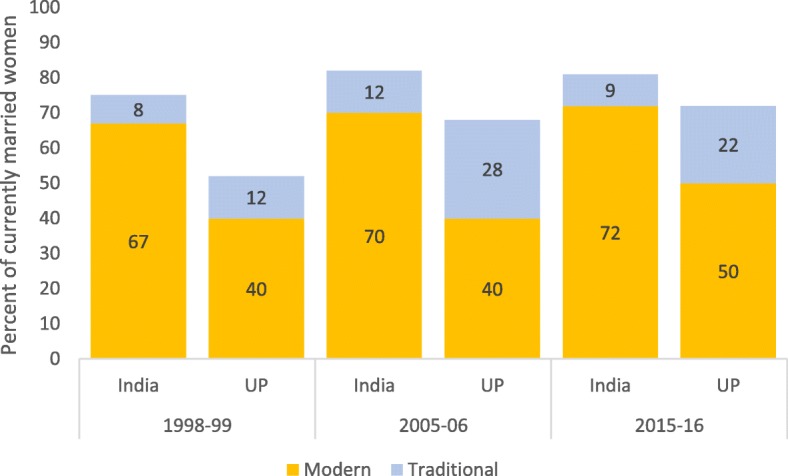
Demand satisfied by modern and traditional methods among currently married women (15–49 years) in India and Uttar Pradesh. Source: National Family Health Survey (NFHS) 1998–99 to 2015–2016

Family planning coverage, the proportion of married women whose demand for modern or traditional methods was met, surpassed 70% in 2015–16 in UP, a level that was reached in India in 1992–93 (Fig. 3). There remains however a large difference between UP and the national level in the demand satisfied for family planning met with modern methods: 49.9 and 72.0% respectively as per NFHS-4.

### Fertility differentials within Uttar Pradesh

**Fig. 4:**
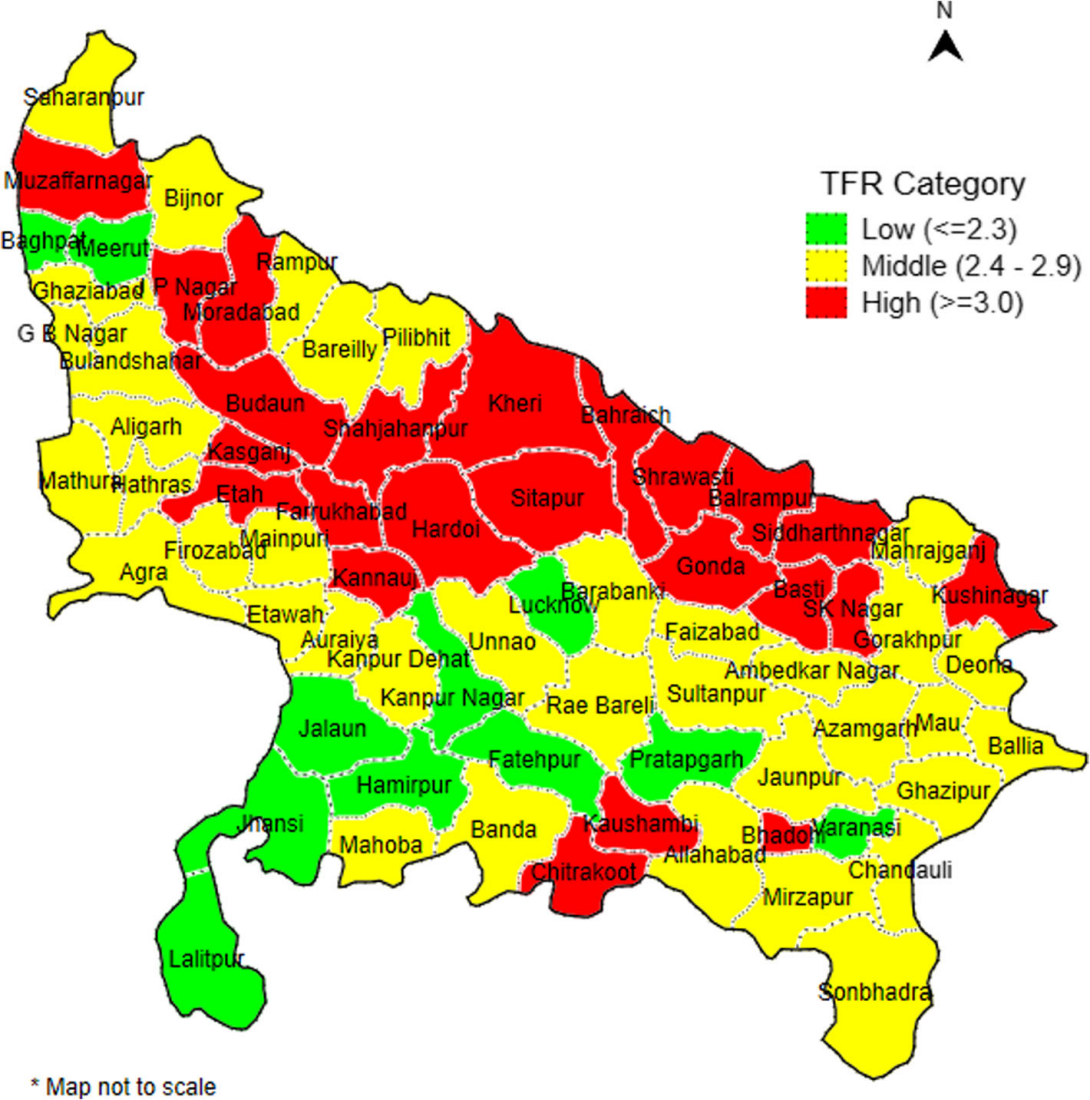
Total fertility rate by district, Uttar Pradesh. Source: National Family Health Survey (NFHS), 2015–16

In 2015–16, the district levels of total fertility ranged from 1.6 in Lucknow and Kanpur Nagar districts; both highly urbanized, in central UP to 4.2 and 4.4 in Shrawasti and Bahraich districts from northern UP bordering Nepal (Fig. 4). One-third of the 71 districts had total fertility levels of 3.0 or higher. Most high TFR districts were clustered in Tarai region (northern-UP) and their adjacent districts. Districts with low TFR were largely located in the southern part of UP (around Bundelkhand region). Several district characteristics were associated with higher total fertility rates including districts with higher proportion of women who did not attend school, low levels of urbanization, larger proportion of the population who are Muslim and greater level of poverty in the district based on bivariate regression results (Table 1); but Scheduled Caste did not have a significant effect. In the multivariate analysis only schooling levels were significantly associated with TFR in 2015–16 (B = 0.6319 *p* < 0.001) and explained more than 60% of the variation in TFR.

**Table 1:** Factors affecting TFR level in 2015–16 and the relative change in TFR during 1998 to 2016

District characteristics	Levels of TFR 2015–16	
% of Currently married women who were:	Bivariate model:	Multivariate model:
	B-coefficient (Std.Error; *p*-value)	B-coefficient (Std.Error; *p*-value)
Not attended school	0.040 (0.004; 0.000)	0.034 (0.007; 0.000)
Urban residents	−0.014 (0.003; 0.000)	−0.001 (0.004; 0.807)
Muslim	0.011 (0.005; 0.030)	−0.000 (0.005; 0.932)
Schedule caste	−0.004 (0.010; 0.674)	−0.007 (0.007; 0.348)
Poor wealth quintile	0.015 (0.003; 0.000)	0.005 (0.005; 0.349)
Model fit (R^2^)		**0.6319**
Among currently married women in districts:	TFR Change 1998–2016	
% Change in women with schooling	0.655 (0.144; 0.000)	0.559 (0.179; 0.003)
% Change in urbanization level	0.110 (0.096; 0.258)	−0.155 (0.098; 0.118)
% Change in Muslim population	−0.175 (0.128; 0.177)	−0.053 (0.119; 0.658)
% Change in Schedule Caste population	−0.258 (0.189; 0.177)	−0.164 (0.173; 0.347)
Baseline level of fertility	10.059 (2.032; 0.000)	7.332 (2.085; 0.001)
Model fit (R^2^)		**0.3865**

Note: Poor wealth quintile includes both poor & poorest quintile

Model fit (R^2^) for both the regressions are statistically significant at *p*<0.001

**Fig. 5:**
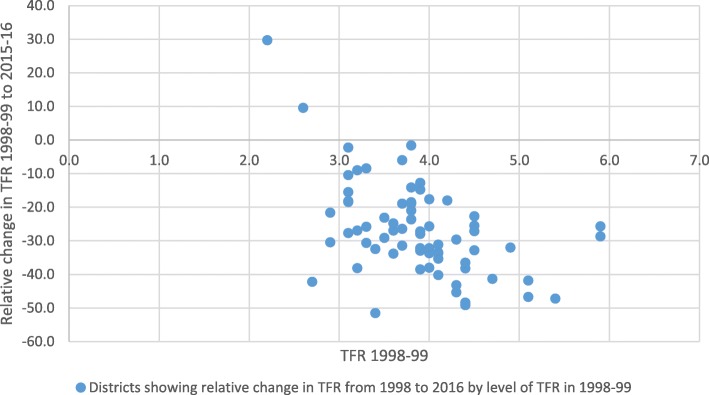
Relative change in district TFR between 1998 & 2016 by level of total fertility in 1998, UP. Source: National Family Health Survey

In the 71 districts with comparable data in NFHS-2 and NFHS-4, fertility declined during the two survey periods in all but two districts. Districts with higher levels of fertility in 1998–99 achieved a greater absolute and relative decline than districts with lower fertility in 1998–99, leading to convergence (Fig. 5).

The relative decline in fertility between 1998 and 2016 was faster among districts where there was an increase in schooling among currently married women and higher baseline TFR level (Table 1). An increase in the proportion of currently married women who were Muslim and belonged to Scheduled Caste did not show a significant association with the size of the decline (Table 1). The multivariate analysis showed that only schooling and the baseline fertility level were statistically significant in explaining relative decline in TFR (Table 1).

### Family planning within Uttar Pradesh

**Fig. 6:**
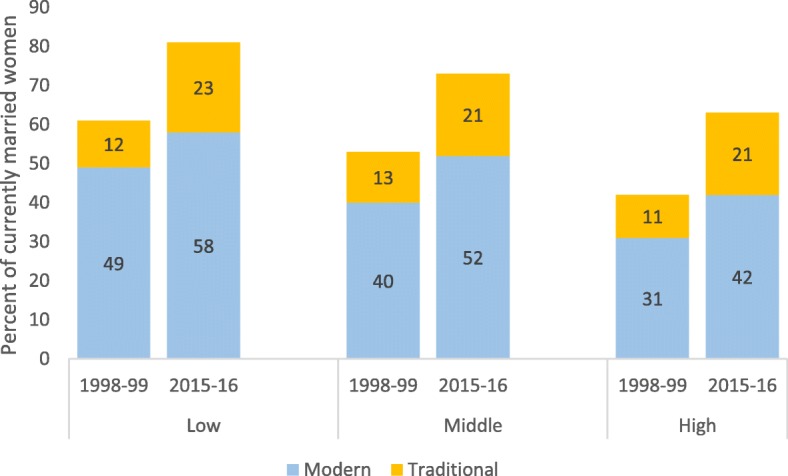
Demand satisfied by modern and traditional methods among currently married women (15–49 years), by current TFR (2015–16). Source: National Family Health Survey (NFHS) 1998–99 and 2015–16

There are considerable differences in levels of family planning coverage and trends of modern methods usage among the three groups of districts classified by the level of fertility (Fig. 6). The demand satisfied by traditional methods increased substantially in all the three groups of districts during 1998–99 to 2015–16 and the proportion of the demand met by traditional methods showed a similar pattern in the three groups of districts, with an increase from 11 to 13% in 1998–99 to 21–23% in 2015–16. The demand satisfied with modern methods also increased by similar absolute proportions in the three groups of districts, but coverage levels still differed markedly in 2015–16 with 58, 52 and 42% in the low, middle and high fertility districts respectively (Fig. 6).

### Range- low: TFR 2.3 or less, middle TFR 2.3–2.9 and high: TFR 3.0 or higher

**Fig. 7:**
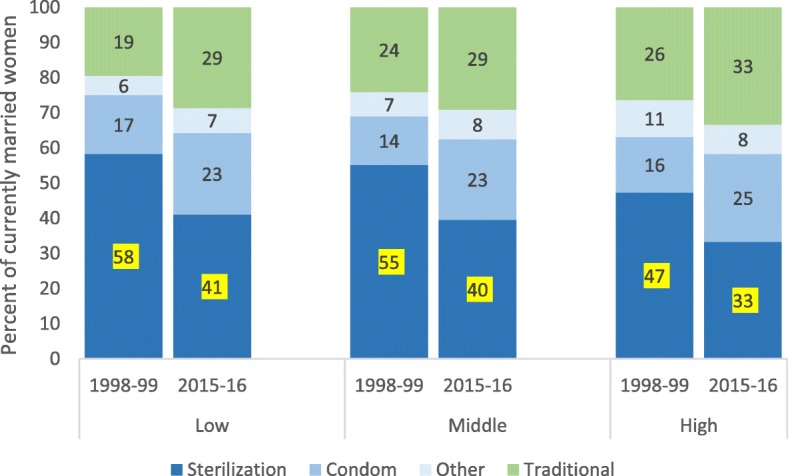
Percent distribution of main contraceptives methods among currently married women (15–49 years), by current TFR (2015–16). Source: National Family Health Survey (NFHS) 1998–99 and 2015–16

The mix of family planning methods has remarkable similarities in the three groups of districts, including the predominance but declining importance of sterilization, the increasing share of the second most common modern method - condoms, the relatively small role of all other modern contraceptives with little increase over time and the increasing proportion of women that rely on traditional methods (Fig. 7). The share of sterilization was 41 and 33% in the low- and high fertility district groups respectively. The share of condoms increased in all three district groups from 14 to 17% in 1998–99 to 23–25% of methods in 2015–16. Traditional methods accounted for 29% of users in low and middle- fertility districts and for 33% in high-fertility districts in 2015–2016.

Instead of clustering the districts based on TFR levels in NFHS-4 as above, we also examined the change of TFR, factors affecting change of TFR and the change in contraceptive use patterns and trends in each district. The new figures have been included in the Appendix. The association between rate of change of TFR and contraceptive usages remain the same. This is primarily because many of the districts with slow rate of change are falling in the same categories of district with Low TFR. Similarly, the districts with faster change are those with high TFR. Though the analysis based on the rate of change of TFR adds another dimension to the findings, it does not change the results substantially.

## Discussion

UP has made an important contribution to India’s fertility decline, particularly since 2000 when UP had a stronger fertility decline than India as a whole. Yet, UP’s total fertility is still 2.7, and 20% above the national level. India’s efforts to meet its fertility target of replacement level largely depends on further fertility decline in large population state such as UP. For instance, 25% of the family planning total unmet need of India is accounted by UP with 18% of India’s population [2].

The fertility decline in UP was driven by an increase in use of contraceptive methods among married women. There are, however, important differences with India in overall patterns of contraceptive use. By 2015–16, female sterilization was the predominant method in UP but sterilization among currently married women was only half as prevalent as in India. Condom use was the second most popular method and increasing, and twice as prevalent as in India. The use of traditional methods of family planning, most notably periodic abstinence, was more than two times higher in UP compared to India.

A survey conducted in 75 districts at facility level in 2018 suggests that supply issues play an important role. For instance, the availability of family planning services in all District Hospitals, Community Health Centers and Block Primary Health Care Centers in all 75 districts of UP indicated that 25% of the facilities provide female sterilization services, 11% male sterilization and 83% provide condoms (India Health Action Trust: Facility mapping for strengthening family planning services in Uttar Pradesh, Unpublished). According to a 2016 survey on family planning practices among currently married women (aged 15–49) conducted in 25 districts of UP, the most preferred method among women who were not using any contraceptive method at the time of survey but intended to use family planning in the future was female sterilization (33%), followed by injectable (22%) and traditional methods (18%) [20]. It was estimated that only 36% of the potential additional users of female sterilization can be provided services with the current system [20]. Reliance on traditional methods of contraception in UP is likely to be associated with higher contraceptive failure rates, contributing to higher fertility and possibly increase in induced abortions. For instance, 16% of women were traditional method users who have completed their desired family size and they all had the unmet need for modern contraceptives [20].

UP has markedly lower family planning coverage with modern methods among currently married women compared to India (50 and 72% in UP and India respectively), indicating that there is a need for major improvements in access to family planning methods (NFHS-4). The family planning program in UP received a boost by the establishment of an autonomous body in the early nineties and, more recently, of a technical support unit for family planning. During the last two decades, the state government has introduced several interventions such as community-based distributors for contraceptives and clinical outreach camps in low-resource settings. These actions might have contributed to the increase in demand satisfied thereby reducing gap with India in the past two decades, but much more is needed.

Within UP there is considerable variation between districts in fertility, ranging from 1.6 to 4.4 in 2015–16, including one-third of districts with fertility levels of 3.0 or higher. Higher fertility districts were more common in the Tarai region and their adjacent districts bordering Nepal, which is considered least developed region in the state. Lower fertility districts were more likely urbanized with better literacy rate.

The decline in UP has not been uniform and there are substantial variations in the TFR across districts of UP. The largest declines in TFR were recorded in districts with higher fertility rates, leading to a convergence of fertility between districts. Furthermore, districts that made greater progress in reducing illiteracy had the greatest fertility decline, irrespective of other district economic or sociocultural characteristics. The trends in coverage of family planning and methods mix were dependent on the level of fertility in the districts, but coverage gaps and differential use of sterilization persisted. The increases in demand satisfied and major increases in the share of traditional methods and condoms among married women were observed in the low, middle and high fertility groups of districts (NFHS-4).

The increase in traditional methods, mainly the rhythm method, is remarkable. The fact that rhythm method and condoms are among the most popular methods used by couples for contraception in UP and the family planning demand met by any method in all the three clusters is quite high especially in the low fertility districts, indicating that couples might be practicing them correctly and should have contributed for fertility reduction. Moreover, as per NFHS-4, there is a stark difference in UP between rural and urban populations in condom use, which was higher among the urban population with almost 20% condom users compared to their rural counterpart of only 8%. Interestingly, in the early nineties when fertility was high, all the three groups of districts had similar levels of demand satisfied. The recent family planning survey indicates that traditional methods are their preferred method of choice along with injectable [20]. Hence, the effectiveness of traditional methods and condom use in avoiding unintended pregnancies in UP cannot be rejected without further investigation.

Our study has a number of limitations. We did not analyse the role of sex preference in fertility differentials. Son preference has been identified as an important factor influencing the desire for more children [21]. In 2015–16, national current use of contraceptives was marked lower among married women with two daughters (53%) compared to those with one or two sons (68–71%) [2]. In UP, the desire for no more children was 84% among currently married women with two boys and no daughters, 77% among those with one boy and one girl and 37% among those with two daughters [22]. Further work to assess the role of son preference in explaining district differences in fertility and family planning use may throw further light on this issue.

The analysis did not consider changes in other determinants of fertility such as age at marriage or the incidence of abortion. The median age at marriage for women increased from 17.1 to 19.0 years in India during 1998–99 to 2015–16, and from 16.3 to 18.5 years in UP. It is unlikely that marriage changes played a major role in limiting fertility, as the median age at marriage is still very low. One of the possible explanations in controlling fertility is rapid increase in induced abortions especially self-administration of medical abortion drugs. A recent study argued that of the estimated 48.1 million pregnancies, 54% resulted in live births, 33% ended in induced abortions and remaining were miscarriages [10]. The authors concluded that facility-based abortions in the same period as NFHS-4 is five times more than the number that was reported to government sources. We did not have data on the supply of family planning commodities, which would give us greater insights into why the coverage of modern family planning methods is inadequate and why traditional methods are so popular. Finally, our analysis did not go beyond the district level. Further insights for better targeting of interventions can be gained from analysis at the sub-district level (blocks).

## Conclusions

This analysis provides valuable insights into fertility and family planning within UP. Major declines in fertility have occurred but much remains to be done to achieve fertility targets, reducing inequalities between districts and ensure that couples have universal access to family planning methods. Given UPs large population, activities need to be appropriately scaled and targeted according to need and geography to ensure the achievement of state-level improvements in family planning programs and fertility outcomes.

## Figures and Tables

**Fig. 8:**
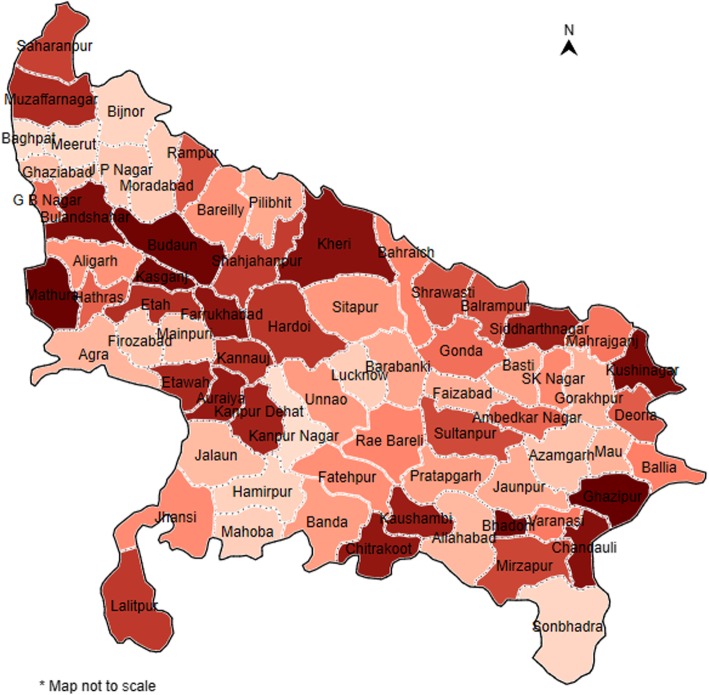
Change in TFR by district, Uttar Pradesh. Note: Light shade denotes faster decline in TFR. Source: National Family Health Survey (NFHS) 1998–99 and 2015–16

**Fig. 9:**
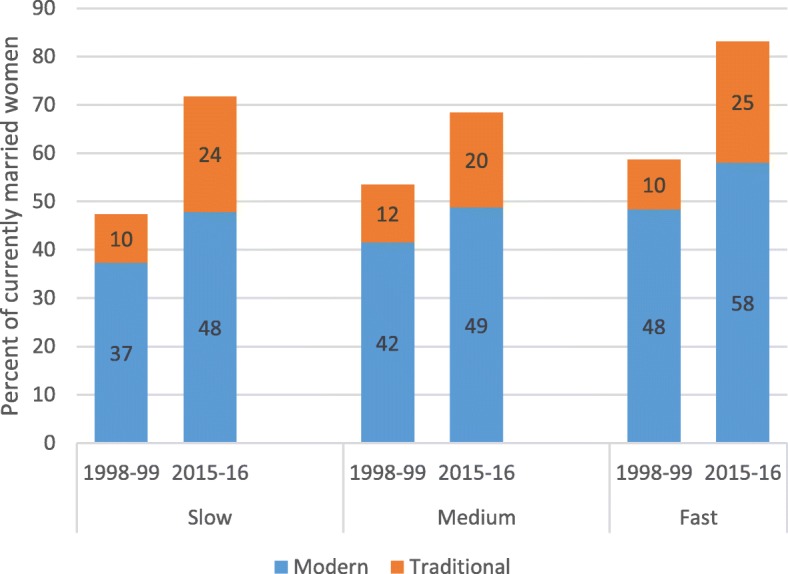
Demand satisfied by modern and traditional methods among currently married women (15–49 years), by change in TFR (1998–99 to 2015–16). Source: National Family Health Survey (NFHS) 1998–99 and 2015–16

**Fig. 10:**
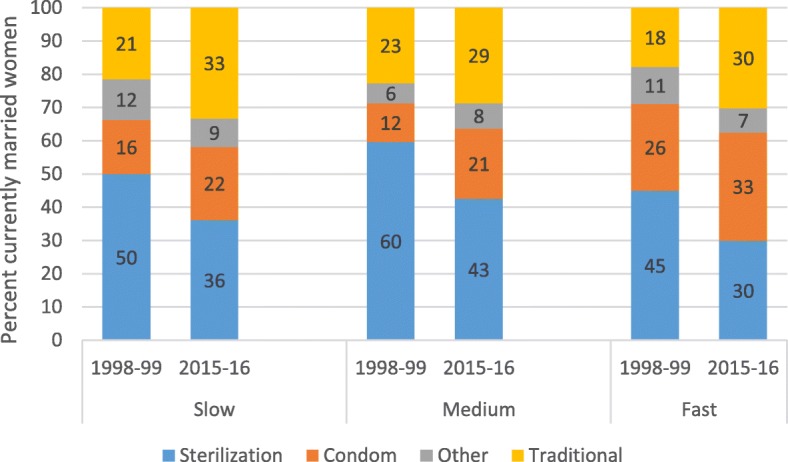
Percent distribution of main contraceptives methods among currently married women (15–49 years), by change in TFR (1998–99 to 2015–16). Source: National Family Health Survey (NFHS) 1998–99 and 2015–16

## Data Availability

Data and material are publicly available for researchers.

## Abbreviations

NFHS: National Family Health Survey
SRS: Sample Registration System
TFR: Total Fertility Rate
UP: Uttar Pradesh
